# Rates of metabolic syndrome in Queensland adult community mental health consumers with schizophrenia and related disorders: A brief report

**DOI:** 10.1177/10398562251358760

**Published:** 2025-07-04

**Authors:** Mike Trott, Sally Plever, Mellisa Anzolin, Irene McCarthy, Dan Siskind

**Affiliations:** Metro South Addiction and Mental Health Services, Metro South Health, Brisbane, QLD, Australia; University of Queensland Faculty of Medicine, 1974 The University of Queensland, Brisbane, QLD, Australia; 90131 Queensland Centre for Mental Health Research, The Park Centre for Mental Health Treatment Research and Education, Wacol, QLD, Australia; Qld Mental Health Clinical Collaborative, Metro North Mental Health, Brisbane, QLD, Australia; NHMRC Centre for Research Excellence on Achieving the Tobacco Endgame, School of Public Health, 1974 The University of Queensland, Brisbane, QLD, Australia; Qld Mental Health Clinical Collaborative, Metro North Mental Health, Brisbane, QLD, Australia; Metro South Addiction and Mental Health Services, Metro South Health, Brisbane, QLD, Australia; University of Queensland Faculty of Medicine, 1974 The University of Queensland, Brisbane, QLD, Australia; 90131 Queensland Centre for Mental Health Research, The Park Centre for Mental Health Treatment Research and Education, Wacol, QLD, Australia

**Keywords:** metabolic syndrome, schizophrenia, monitoring

## Abstract

**Objective:**

People with schizophrenia are at increased risk of metabolic syndrome (MetS), contributing to excess morbidity and mortality. This study examined MetS monitoring rates and prevalence in people with schizophrenia receiving public mental health care in Queensland.

**Methods:**

Data from the Consumer Integrated Mental Health and Addiction Application (CIMHA) were extracted for individuals aged 18–64 with a schizophrenia diagnosis. MetS was determined using International Diabetes Federation criteria.

**Results:**

Of 5802 individuals, 16.0% had sufficient data to determine MetS status. Among those with complete data, MetS prevalence was 53.2%. MetS was significantly more common in regional than metropolitan areas. Blood pressure and BMI were recorded for most patients (≥74%), but biochemical indices (fasting glucose, HDL, triglycerides) were recorded in only 26.4%–35.5%.

**Conclusions:**

MetS is highly prevalent in schizophrenia, yet routine monitoring is incomplete, particularly for biochemical markers. Strengthening data integration across healthcare systems and ensuring access to evidence-based interventions for MetS management, particularly in regional areas, is critical to addressing this major health disparity.

People experiencing mental illness have 15–20 years reduced life expectancy, largely attributable to preventable illnesses such as cardiovascular and metabolic disorders.^1–3^ The Lancet Psychiatry Commission’s blueprint for protecting the physical health of people with mental illness identified that people with physical and psychiatric comorbidity have higher hospital costs, increased readmission rates, and higher total health costs compared to the general population.^4^ People experiencing mental illness are vulnerable to cardiometabolic disturbances, including metabolic syndrome (MetS), a clustering of cardiovascular risks that increase the likelihood of developing type-2 diabetes.^5^

Studies have identified people with schizophrenia are at high-risk of MetS. For example, in a Queensland sample of psychiatric patients treated with clozapine (prescribed for treatment-resistant schizophrenia), 45% had MetS, yet less than half had complete MetS indices recorded in their medical record.^6^ In Queensland, people treated with antipsychotic medications undergoing treatment in community mental health services receive routine six-monthly physical health assessments which, during 2012 to 2018, included recording of physical health indices via an electronic Metabolic Monitoring Form (MMF) and/or a physical health assessment code within the Consumer Integrated Mental Health and Addiction Application (CIMHA: the Queensland mental health database), completed by any healthcare professional undertaking the assessment A 2018 review of medical records for the documentation of physical health assessment in people with schizophrenia and related disorders demonstrated a statewide average of 68% completion.^7^ Completion of MetS indices, however, was not reviewed, and it is likely that completion in people with schizophrenia may be lower than for a population of clozapine users. The systematic capturing of metabolic information presents a unique opportunity to examine MetS prevalence in people with schizophrenia who are receiving care in public mental health services. The aim of this study, therefore, was to examine monitoring rates and prevalence of MetS in people with schizophrenia in Queensland, and to explore determinants of non-complete monitoring and MetS prevalence.

## Methods

### Data preparation and extraction

Ethics approval was provided by the Metro North Human Research and Ethics Committee (HREC/2023/MNHA/101747).

Data was extracted from CIMHA, with consumers included if they were 18–64 years and had a diagnosis of schizophrenia based on ICD-10-AM codes (F20, F23, and F25 disorders). Consumers were excluded if they were primarily treated within acute inpatient/extended care facilities, correctional units, or under consultation liaison services. The scope of this analysis was all consumers during the 2018 calendar year that had a MMF completed. If consumers had >1 MMF form during the reference period, priority was given to the form with more complete information on the MetS parameters defined by the International Diabetes Federation (IDF).^5^ In brief, MetS was determined if consumers had indicators of central obesity and any two of raised triglycerides; HDL; blood pressure; or fasting plasma glucose. Because consumer country/ethnic group was unavailable, Europid thresholds for waist circumference were used.^8^ In addition, if waist circumference was unavailable, a BMI of >30 kg/m^2^ was used as an indicator of central obesity, see Table 1. If several forms had the same number of parameters, the earliest completed form was used. Extracted data included BMI; waist circumference; blood pressure; fasting glucose; high density lipoproteins; and triglycerides. Location of service was dichotomised into metropolitan (population >500,000) or regional locations. The IDF criteria was used for the determination of MetS, however

**Table 1.** Criteria for classification of metabolic syndrome.

Central obesity	Waist circumference ≥94 cm for males and ≥80 cm for females^a^
	OR
	If waist circumference data was unavailable, a BMI of >30 kg/m2 was used as an indicator of central obesity
And any two from:	
Raised triglycerides	≥150 mg/dL (1.7 mmol/L)
Reduced HDL cholesterol	<40 mg/dL (1.03 mmol/L) in males or <50 mg/dL (1.29 mmol/L) in females
Raised blood pressure	Systolic ≥130 or diastolic ≥85 mm Hg, or treatment of previously diagnosed hypertension
Raised fasting glucose or previously diagnosed type 2 diabetes	≥100 mg/dL (5.6 mmol/L)

HDL: high density lipoprotein.

^a^Because consumer country/ethnic group was unavailable, Europid thresholds for waist circumference were used.

Statistical analyses were conducted in R, version 4.4.0. To determine if variables were associated with (a) enough information to determine MetS, and (b) MetS prevalence, multivariate logistic regressions (adjusted for age and sex) were conducted. Missing data was deleted listwise.

## Results

**Figure 1.**
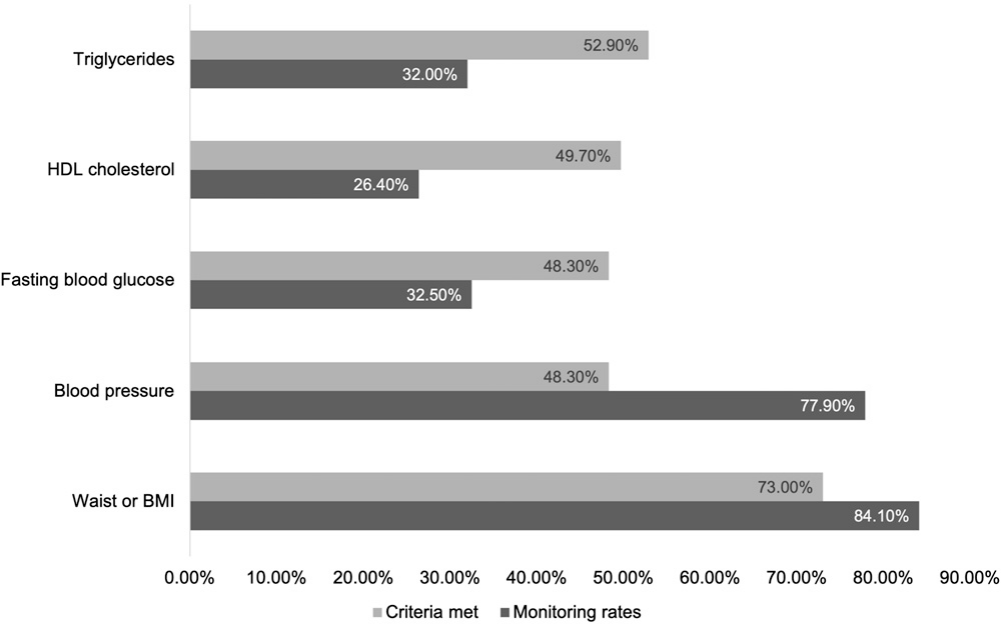
Rates of available monitoring data for metabolic syndrome components and rates of these metabolic syndrome components above the respective IDF thresholds

Of 9562 records, 5802 unique consumers were extracted, of whom 69.7% were male, with a median age = 40.2 (IQR = 16.6). Regarding monitoring of MetS indices, 53.4% had waist circumference, 74.1% BMI, 77.9% blood pressure, 32.5% fasting glucose, 26.4% HDL, and 32.0% had triglycerides information. Of these, 73.0%, 48.3%, 48.3%, 49.7%, and 52.9% were above the respective IDF thresholds, see Figure 1. A total of 16.0% (*n* = 931) had enough information to determine MetS. There were no significant associations between collected variables and having sufficient data to determine MetS. Of the sub-sample that had sufficient information to determine MetS, MetS prevalence was 53.2% (*n* = 495), and regional services were significantly associated with higher rates of MetS, see Table 2.

**Table 2.** Demographic information and differences between proportions.

		Monitoring rates (*n* = 5802)					Sub-sample only including cases with sufficient information to determine metabolic syndrome status (*n* = 931)				*p-*value
		Total sample	Incomplete information (*n* = 4841)	Complete information (*n* = 961)	aOR	*p*-value	Total sub-sample	No metabolic syndrome^a^ (*n* = 436)	Metabolic syndrome^a^ (*n* = 495)	aOR	
Gender	Male	4046	3393 (83.9%)	653 (16.1%)	1.03 (0.89-1.21)	0.67	653 (70.1%)	319 (73.2%)	334 (67.5%)	0.78 (0.59-1.04)	0.09
	Female	1756	1478 (84.2%)	278 (15.8%)			278 (29.9%)	117 (26.8%)	161 (32.5%)		
Age	Median (IQR)	40.2 (16.6)	40.1 (16.6)	40.8 (16.5)	1.00 (1.00-1.01)	0.20	40.18 (16.6)	40.8 (17.5)	40.8 (15.9)	1.01 (1.00-1.02)	0.22
Location	Metropolitan	3374	2844 (84.3%)	530 (15.7%)	1.07 (0.92-1.23)	0.38	530	269 (50.8%)	261 (49.2%)	1.47* (1.13-1.91)	0.004*
	Regional	2428	2027 (83.5%)	401 (16.5%)			401	167 (41.7%)	234 (58.3%)		

aOR: adjusted odds ratio (adjusted for age and gender).

*indicates significant results *p* ≤ 0.05.

^a^Based on the International Diabetes Federation definition of metabolic syndrome.

## Discussion

This study aimed to examine monitoring rates and prevalence of MetS in schizophrenia, and to explore determinants of these in a Queensland-wide cohort. We found that, where data was adequately recorded, over half of the patients had MetS, while almost three-quarters were overweight/obese. Around half had abnormal results for each of blood pressure, fasting glucose, HDL cholesterol or triglycerides. However, data on all five of these parameters was completed in only 1/6^th^ of forms.

This may reflect challenges in integrating databases that monitor MetS. A 2017 study examining monitoring rates of people taking clozapine in Brisbane, reported that 43% of charts had sufficient MetS data, with similar overall rates of MetS. However, data from the study were taken from (unspecified) chart notes, pathology reports and the MMF.^6^ In our study, frequency of recording data on pathology results (26.4% to 35.5%) was considerably lower than for BMI and blood pressure (74.1% to 77.9%). This may reflect both a lower rate of blood testing and barriers to transcribing blood results. For example, although blood tests are conducted, they may not be recorded on the MMF. In addition, people with schizophrenia may decline medical procedures due to symptoms of psychosis and/or challenges with accessing medical services, including inadequate linkage with primary care.^9^ Our findings highlight the need for electronic monitoring databases for MetS monitoring to be better integrated to allow data sharing. This integration is crucial to allow the creation of clinical decision support systems for managing comorbid physical health disorders among people with severe mental illness.

Regarding MetS prevalence, other Australian studies have yielded similar rates. For example, Tso et al. (2017) yielded a MetS prevalence of 45% amongst clozapine users, whereas Lappin et al. (2018) reported a higher prevalence of 58% amongst a similar population.^10^ Furthermore, Morell et al. (2019) reported a prevalence of 44% amongst people taking injectable antipsychotic medication^11^; whilst a Western Australian study yielded a prevalence rate of 51% amongst people with schizophrenia, and also reported that age and gender were not significantly associated with risk,^12^ which broadly concurs with our results. Of note, we found higher prevalence of MetS outside metropolitan areas, concurring with other MetS studies in schizophrenia.^13^ The high prevalence of MetS in people with schizophrenia is concerning, particularly because it is a key driver of the large mortality gap. It highlights the need to ensure that we are not just monitoring for MetS, but providing evidence-based interventions for treatment, particularly outside metropolitan centres.^1^

The results of this study should be considered within its limitations. Importantly, several demographic and geographic factors were not available. While we were able to include a larger sample than other Australian studies, we were only able to access the monitoring information recorded in one form. Furthermore, the dataset was from 2018, the last year for which complete data was available. Requirements changed during COVID, and current data is not available. As such, it may not represent current trends.

In conclusion, a concerningly high proportion of people with schizophrenia in Queensland have MetS. Of note, there are higher rates of MetS outside metropolitan areas. Monitoring alone is insufficient. This finding highlights the need to ensure that evidence-based lifestyle/pharmacological interventions are offered to people living with schizophrenia with comorbid MetS.

## Data Availability

Due to privacy, ethical and legal considerations, this data cannot be shared without direct approval from data custodians and the Office of Research and Innovation of Queensland Health. Contact details for Queensland Health custodians can be found at https://www.health.qld.gov.au/__data/assets/pdf_file/0034/843199/data_custodian_list.pdf
